# Cannabinoid Therapies in Less-Common Disorders: Clinical Evidence and Formulation Strategies

**DOI:** 10.3390/diseases14020083

**Published:** 2026-02-23

**Authors:** Silvia Afonso, Joana Gonçalves, Ana T. Brinca, Luana M. Rosendo, Tiago Rosado, Ana Paula Duarte, Eugenia Gallardo

**Affiliations:** 1 Faculty of Health Sciences, Department of Medical Sciences, University of Beira Interior, Avenida Infante D. Henrique, 6200-506 Covilhã, Portugal; silvia.afonso@ubi.pt (S.A.); anabrinca99@gmail.com (A.T.B.); may.rosendo@ubi.pt (L.M.R.); tiago.rosado@ubi.pt (T.R.); apduarte@fcsaude.ubi.pt (A.P.D.); 2 Centre for the Research and Technology of Agroenvironmental and Biological Sciences, CITAB, Inov4Agro, University of Trás-os-Montes and Alto Douro, UTAD, Quinta de Prados, 5000-801 Vila Real, Portugal; 3 RISE-Health, Faculty of Health Sciences, Department of Medical Sciences, University of Beira Interior, Avenida Infante D. Henrique, 6200-506 Covilhã, Portugal; 4 Laboratory of Pharmacotoxicology, UBIMedical, University of Beira Interior, EM506, 6200-000 Covilhã, Portugal; 5 Beiras Academic Clinical Center (CACB)—Group of “Problemas Relacionados com Toxicofilias”, UBIMedical, EM506, 6200-000 Covilhã, Portugal

**Keywords:** cannabinoids, medical cannabis, rare and complex disorders, clinical evidence, drug delivery systems, safety and regulation

## Abstract

**Background/Objectives:** Cannabinoids are increasingly recognised for their therapeutic potential beyond well-established indications such as chronic pain, multiple sclerosis, and specific epileptic syndromes. Recent advances have highlighted their possible role in less-common or orphan diseases, opening new avenues for pharmaceutical research and clinical application. **Methods:** This review provides a critical synthesis of the most recent evidence (2020–2025), available in PubMed and Scopus, regarding the use of cannabinoids in conditions including refractory epilepsies beyond Dravet and Lennox–Gastaut syndromes, movement disorders such as dystonia and Tourette syndrome, rare dermatological diseases like epidermolysis bullosa, and emerging data in Crohn’s disease. **Results:** Negative outcomes, such as those reported in Fragile X syndrome trials, are also discussed as instructive examples of methodological and pharmacological challenges. Particular attention is given to the optimisation of pharmaceutical formulations and advanced separation technologies, including oromucosal sprays, transdermal gels, and novel nanocarrier systems, which aim to overcome issues of bioavailability and variability in patient response. Finally, safety concerns, regulatory aspects, and the need for robust clinical trials are addressed. **Conclusions:** Overall, cannabinoids represent a promising yet underexplored therapeutic option in rare and complex disorders, warranting further investigation supported by innovative pharmaceutical approaches.

## 1. Introduction

Cannabinoids have gained increasing recognition as therapeutic agents over the past two decades, driven by advances in pharmacology, regulatory changes, and a growing body of clinical evidence supporting their medical use [1,2]. While cannabis-derived products have historically been associated with recreational consumption, contemporary research has progressively repositioned cannabinoids as pharmacologically relevant compounds with well-defined mechanisms of action mediated primarily through the endocannabinoid system [3,4]. This system plays a central role in the modulation of pain perception, neuroinflammation, immune responses, motor control, and gastrointestinal function, providing a strong biological rationale for therapeutic intervention across a range of clinical conditions [1,5].

At present, the medical use of cannabinoids is largely confined to a limited number of well-established indications. For example, in Portugal, medicinal cannabis is authorised by the National Authority of Medicines and Health Products (Infarmed) for specific conditions, including chronic pain associated with oncological or neurological disease, spasticity related to multiple sclerosis or spinal cord injury, chemotherapy-induced nausea and vomiting, appetite stimulation in palliative care, treatment-resistant glaucoma, Tourette syndrome, and severe childhood epilepsies such as Dravet and Lennox–Gastaut syndromes [6]. These approved indications reflect areas in which clinical efficacy has been demonstrated with sufficient consistency to justify regulatory acceptance, particularly through standardised cannabis-based preparations and purified cannabidiol formulations [2,7]. Nevertheless, they also highlight the relatively narrow therapeutic scope within which cannabinoids are currently prescribed, despite their broad pharmacodynamic profile and multisystem effects [7,8]. In parallel with these approved uses, there has been a marked expansion of experimental and off-label investigations exploring cannabinoid-based therapies in less-common, rare, or complex disorders [9,10]. Many of these conditions are characterised by chronic symptom burden, limited treatment options, and substantial impact on quality of life, often meeting criteria for orphan disease designation [10]. In such contexts, conventional pharmacological strategies frequently provide inadequate symptom control or are associated with significant adverse effects, creating a pressing need for alternative or adjunctive therapeutic approaches. Cannabinoids, particularly non-psychoactive compounds such as cannabidiol (CBD), have emerged as promising candidates due to their multimodal mechanisms, favourable tolerability profiles, and potential to modulate neuroinflammatory, neuromodulatory, and immune pathways [1,5].

Despite growing clinical interest, the translation of cannabinoid research into routine clinical practice for rare or less-common disorders remains challenging [10]. Evidence is often fragmented, derived from small clinical trials, observational studies, or heterogeneous patient populations, and outcomes are frequently variable [11]. In addition, cannabinoids present well-recognised pharmaceutical challenges related to poor aqueous solubility, variable bioavailability, extensive first-pass metabolism, and marked inter-individual pharmacokinetic variability [12]. These limitations have prompted the development of innovative formulation strategies, including oromucosal sprays, transdermal systems, and nanocarrier-based delivery platforms, aimed at improving absorption, reducing variability, and enhancing therapeutic consistency. The optimisation of formulation and route of administration is therefore a critical determinant of clinical success, particularly in vulnerable populations and rare disease settings [12].

Safety considerations and regulatory frameworks further complicate the clinical adoption of cannabinoid-based therapies. Although cannabinoids are generally well tolerated, their interaction with cytochrome P450 enzymes, potential for drug–drug interactions, and context-dependent adverse effects necessitate careful clinical monitoring [12,13]. Moreover, regulatory acceptance varies substantially between jurisdictions, reflecting differences in risk–benefit assessment, evidentiary standards, and historical perceptions of cannabis-derived products [1,8]. These factors underscore the importance of critically appraising both positive and negative clinical outcomes, as well as identifying methodological limitations and unmet research needs.

Against this background, the present review aims to provide a comprehensive and critical synthesis of the most recent clinical evidence published between 2020 and 2025 regarding the use of cannabinoids in less-common and emerging clinical indications. Particular emphasis is placed on disorders that fall outside currently approved therapeutic uses, including rare neurological, dermatological, gastrointestinal, psychiatric, and sleep-related conditions. In addition, this review examines contemporary formulation strategies designed to overcome pharmacokinetic limitations, discusses safety and regulatory considerations, and highlights key gaps in current knowledge. By integrating clinical evidence with pharmaceutical and regulatory perspectives, this article seeks to clarify the realistic therapeutic potential of cannabinoids in rare and complex disorders and to inform future research and clinical decision-making. In this review, terminology reflects the nomenclature used in the original studies. “Medicinal cannabis” generally refers to whole-plant preparations or extracts, “phytocannabinoids” to plant-derived compounds such as Δ9-tetrahydrocannabinol (THC) and cannabidiol (CBD), and “cannabinoid therapies” as an umbrella term encompassing plant-derived, synthetic, or purified cannabinoid-based interventions. Where possible, the specific compound or formulation evaluated in each study is explicitly indicated.

## 2. Overview of Cannabinoids and the Endocannabinoid System

### 2.1. Brief Description of the Main Cannabinoids

**Figure 1:**
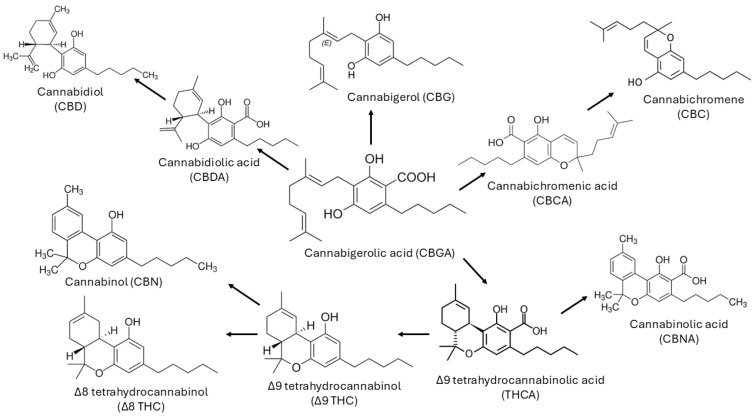
Main cannabinoids present in *Cannabis sativa* L.

Terpenophenol cannabinoids are the most representative class of bioactive compounds present in Cannabis, with trans-Δ-9-tetrahydrocannabinol (THC) being the most potent in terms of psychoactive activity, among the more than 100 cannabinoids identified to date [14,15]. The (-)-trans isomer occurs naturally, although four stereoisomers of THC are known [16]. Other cannabinoids present in Cannabis include tetrahydrocannabinolic acid (THCA), cannabinol (CBN), CBD, and cannabidiolic acid (CBDA) [15]. Cannabigerolic acid (CBGA) is the precursor of CBDA and THCA [15]. When burned, it gives rise to THC and can also give rise, albeit in smaller quantities, to Δ-8-tetrahydrocannabinol (Δ8-THC) [15,16]. On the other hand, CBN results from the oxidative degradation of THC, especially in aged Cannabis samples [17,18,19,20] (Figure 1).

The legalisation of cannabis for medicinal purposes is growing, and several cannabinoids have been identified in cannabis strains. The main types of natural cannabinoids belong to the following families: cannabinol, cannabigerol (CBG), cannabitriol, cannabichromene, cannabinodiol, CBD, isocannabinoids, tetrahydrocannabinol, cannabicyclol, cannabielsoin, cannabicitran and cannabichromanone [16]. However, in these cases, the composition of the samples must be characterised by a specific composition, with reduced levels of THC (0.2–0.3% *w*/*w*) and with CBD and CBDA as major compounds [17,18,19]. The most promising compound for therapeutic purposes is CBD [21]. This compound has demonstrated several beneficial pharmacological effects, notably in combating inflammation, diabetes, cancer and affective or neurodegenerative diseases [21]. More recently, Δ-9-tetrahydrocannabivarin (THCV) has also shown potential use in the treatment of obesity [22]. Even so, Cannabis samples for use in the pharmaceutical and nutraceutical fields have CBDA as the most abundant compound [15].

### 2.2. Relevant Mechanisms of Action

The endocannabinoid system is the primary pathway through which cannabinoids exert their physiological and therapeutic effects. The action of these compounds occurs mainly through cannabinoid receptor type 1 (CB1) and cannabinoid receptor type 2 (CB2) receptors via mechanisms of agonism, modulation or antagonism [23]. These receptors are found coupled to Gi/Go proteins, leading to the inhibition of adenylate cyclase and, consequently, to the reduction of the conversion of AMP to cyclic AMP [23]. The diversity of pharmacological responses is largely linked to the fact that these receptors have a wide distribution in the body [16]. CB1 receptors are found primarily in the central and peripheral nervous system, but also in organs such as the heart, spleen, endocrine glands, and tissues of the gastrointestinal, urinary, and reproductive systems [24]. On the other hand, CB2 receptors predominate in immune and hematopoietic cells, namely leukocytes, spleen and tonsils, and are of particular therapeutic interest due to their regulatory role in inflammatory processes [23,25].

The affinity and effectiveness of cannabinoids depend on the receptor. In the specific case of THC, there is partial agonism of CB1 and CB2 receptors, being more effective on the former [23,26]. Activation of CB1 receptors explains both its psychoactive and analgesic effects, since these receptors directly modulate nociceptive transmission [17]. At the immunological level, the interaction of THC with CB2 receptors contributes to immunomodulatory effects, influencing inflammatory responses and neuroinflammation processes [17]. Thus, although THC has relevant therapeutic potential, its psychoactive profile and the possibility of inducing anxiety, dysphoria or psychotic symptoms in susceptible individuals limit its wider clinical applicability [27]. Conversely, CBD is widely recognised for its therapeutic properties without significant psychoactive effects. Despite having low affinity for CB1 and CB2 receptors [28,29], CBD exhibits a multi-target pharmacology [30], involving both modulators of the endocannabinoid system and other physiological pathways. Recent studies suggest that CBD may exert its beneficial effects through GPR55 receptors in the CA1 hippocampus [31,32]. Others have shown that CBD acts as an allosteric modulator of the CB1R receptor, indirectly influencing its activity [29]. CBD also non-competitively antagonises CB1 and CB2 agonists [33] and may modulate THC effects under certain circumstances. In addition, this compound inhibits anandamide reuptake as well as its enzymatic hydrolysis [34].

Cannabinoids also act on transient potential channels (TRP) [16]. These, found in cell membranes, interact with different molecules, triggering a response [35]. In the particular case of TRPV1s, which are distributed throughout the dorsal root and trigeminal ganglia, skin, bladder, brain, peripheral nerve endings, pancreas and testes, they are activated by the endocannabinoid anandamide under specific conditions [36]. Studies have shown that CBD also has the ability to stimulate vanilloid receptors (VR1), with effects similar to the natural agonist of this receptor [34]. This receptor is associated with effects such as inflammatory hyperalgesia, whose rapid desensitisation, with subsequent paradoxical analgesic and anti-inflammatory effects, is frequent [37]. However, CBD has been shown to be able to exert anti-inflammatory action by desensitising VR1 [34]. CBD also exerts significant actions on the serotonergic 5-hydroxytryptamine 1A (5-HT_1A_) receptor, acting as an agonist of these receptors [37,38]. This mechanism translates into anxiolytic and neuroprotective effects [39]. Another relevant mechanism is the action of CBD in decreasing adenosine reuptake [37]. This effect may lead to a decrease in inflammation through the inhibition of the reuptake of this compound [37].

**Figure 2:**
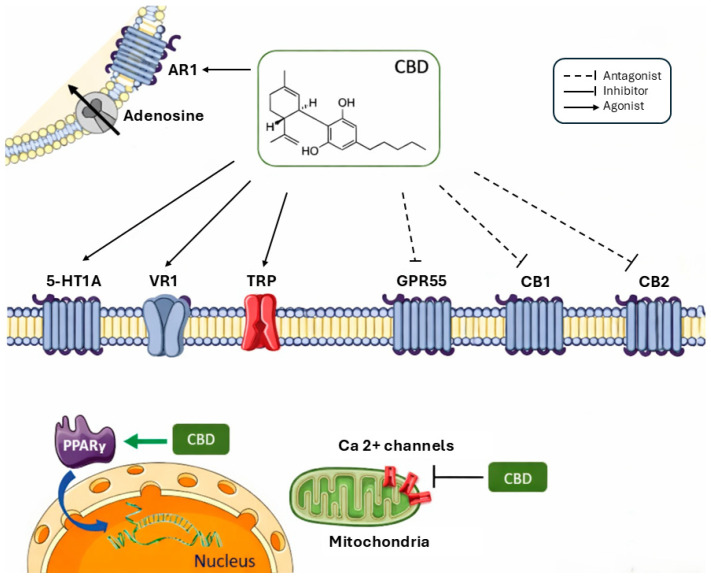
Main mechanisms of action of CBD. CBD acts as an antagonist at GPR55 receptor and as negative allosteric modulator at cannabinoid receptors CB1 and CB2. In addition, CBD acts as an agonist at 5-HT1A, VR1 and other TRP channels, contributing to anxiolytic, anti-inflammatory and analgesic effects. CBD also decreases adenosine reuptake, leading to increased adenosine signalling and reduced inflammation, while acting as a full agonist at AR1 receptors, which may influence cardiac arrhythmias and myocardial injury. Furthermore, CBD acts as an agonist at PPARγ receptors, modulating gene transcription and affecting glucose and fatty acid metabolism. Overall, CBD inhibits calcium channels, a mechanism that may contribute to its potential antiepileptic effects. Legend: A1R, adenosine A1 receptor; CB1, cannabinoid receptor type 1; CB2, cannabinoid receptor type 2; CBD, cannabidiol; GPR55, G protein-coupled receptor 55; PPARγ, peroxisome proliferator-activated receptor gamma; TRP, transient receptor potential channels; VR1: vanilloid receptors; 5-HT1A, 5-hydroxytryptamine receptor 1A.

The modulation of intracellular calcium homeostasis represents another mechanism of particular therapeutic relevance. CBD is able to exert its pharmacological effects by modulating the intracellular concentration of Ca^2+,^ namely by increasing [Ca^2+^]i in hippocampal neurons, more specifically through the release of mitochondrial Ca^2+^ and L-type voltage-gated Ca^2+^ channels [21]. CBD also has a high antioxidant power; however, the increase in [Ca^2+^]i in a tumour environment leads to the formation of reactive oxygen species (ROS) and cell apoptosis [40,41]. In fact, studies have shown that CBD hydroxyquinone reduces colon cancer growth in athymic mice [42]. Furthermore, some cannabinoids activate peroxisome proliferator-activated receptors (PPARs), which consist of groups of nuclear receptor proteins that regulate gene expression, development, metabolism, cell differentiation, and tumorigenesis in higher organisms [16]. Some of the effects associated with the use of cannabinoids are triggered in this way, namely analgesic, antitumor, gastrointestinal, neuroprotective, anti-inflammatory, neuronal function modulation, metabolic and cardiovascular effects [43]. The main mechanisms of action are described in Figure 2.

Although less studied than THC and CBD, other cannabinoids act on the body through these and other mechanisms. The compounds CBG and cannabichromene (CBC) have the ability to inhibit anandamide inactivation, potentially enhancing the effects of endocannabinoids, as they exhibit very low affinity for CB1 and CB2 receptors [40]. CBG and CBC are also capable of activating TRPV1, and along with the latter, CBD, CGB, and CBDA exhibit the ability to activate TRPA1. Additionally, CBD, CGB, and CBDA act as antagonists of transient receptor potential melastatin type 8 (TRPM8) [40,44]. THCV also behaves as a potent partial agonist of CB2 and as an antagonist of CB1 [45]. Due to this dual action, this compound shows therapeutic potential in appetite control [46] or in epilepsy [47].

### 2.3. Formulation Challenges According to General Pharmacokinetic/Pharmacodynamic Processes

Cannabinoids have been increasingly attracting interest in the scientific community; however, the pharmacodynamics and pharmacokinetics of these compounds are not widely known [48]. In the case of cannabinoids with therapeutic applications, the scarcity of data is even more pronounced, limiting the possible applications [48]. Understanding pharmacokinetics is closely linked to the route of administration [16]. According to the literature, the processes of absorption, distribution, metabolism, and excretion exhibit high inter-individual variability, hindering the use of these compounds for medicinal purposes.

The respiratory tract is the preferred route for administering cannabinoids, with vaporisation being used, although not exclusively, for therapeutic purposes [48]. Inhalation allows cannabinoids to pass rapidly from the lungs into the bloodstream and subsequently to the brain, with CBD being detected seconds after inhalation [48,49]. This compound reaches its maximum concentration between approximately 3 and 10 min after consumption; however, its bioavailability is about 31% [23,50,51,52]. Factors such as depth and volume of inhalation, combustion temperature and device used, and compound losses due to pyrolysis lead to great variability [48,53,54,55].

Oral administration in capsule form is also widely used in a therapeutic context, with CBD exhibiting a bioavailability of less than 20%, due to the lipophilicity of the compound and hepatic metabolism [23,25,49,56,57]. Even so, CBD can reach peak concentration in the bloodstream between 1 and 2 h after consumption [23,48]. The oromucosal and sublingual routes are also common in a therapeutic context, allowing not only faster absorption but also avoiding first-pass hepatic metabolism [15,48]. Additionally, plasma concentrations of cannabinoids are higher compared to the oral route [48]. However, cannabinoids have a lipophilic character, which is one of the main obstacles to the formulation of this type of drug. In the case of CBD, it has an oil/water partition coefficient of 6.3, indicating a strong lipid preference [58]. This factor makes it difficult to dissolve in aqueous media, limits oral absorption, and contributes to significant variability in bioavailability [25,56,57]. In fact, studies report a great heterogeneity with respect to the range of concentrations detected, which vary from 0.4 to 16.5 μg/L [59,60]. Additionally, absorption of compounds when ingested orally is also highly influenced by diet, pH of the gastrointestinal tract and type of formulation [58,61]. Concomitant ingestion with foods rich in fat favours absorption (about 14 times) and contributes to the reduction of first-pass hepatic metabolism and lymphatic transport [58]. In the particular case of oromucous and sublingual formulations, these allow bypassing first-pass metabolism; however, a significant portion of the dose is absorbed in the gastrointestinal tract after swallowing, affecting bioavailability [58].

Also, for therapeutic purposes, the transdermal route is used, once again avoiding first-pass metabolism [23,49]. Although cannabinoids have a lipophilic character and, consequently, limited dermal diffusion, CBD permeates the skin more easily than THC [48,62,63]. Once again, this pathway allows bypassing first-pass hepatic metabolism [16]. Studies have shown that the use of gels and emulsions can contribute to an increase in plasma concentrations of CBD, when compared with oil formulations [58]. However, skin permeability remains a challenge and is also dependent on the use of excipients [62,64,65].

Finally, although less studied, both the ophthalmic and rectal routes constitute alternatives for therapeutic application [23]. Other routes of administration for recreational use include smoking and oral administration in the form of food products [16].

The distribution of cannabinoids throughout the body’s tissues occurs rapidly, so their plasma concentration decreases in the same way [49]. Some factors, such as the chemical properties of the molecules, the vascularisation of the tissues, the body composition and the health status of each individual, are closely linked to the degree of distribution of these compounds [66]. Thus, more vascularized tissues, such as the brain, lungs, heart, and liver, rapidly accumulate these compounds, with a volume of distribution for CBD of 32 L/kg [51,67,68,69,70]. The lipophilicity of cannabinoids favours their accumulation in adipose tissue, which can result in prolonged redistribution [23,48,51,67]. This accumulation can greatly influence elimination times, which can vary from hours to weeks. This fact could explain the persistence of cannabinoids in the body. Additionally, individual factors such as sex, body composition, or variations in metabolism can also influence tissue and plasma concentrations [23,66]. Once again, these factors contribute significantly to the difficulties in obtaining formulations with controlled release over time, especially in repeated doses.

After distribution, CBD undergoes metabolism in the liver, initially by CYP2C19 and CYP3A4 and subsequently by CYP1A1, CYP1A2, CYP2C9 and CYP2D6 [13,48]. Even though the metabolization reactions include oxidations at C-9 and in the side chain, a portion is excreted unchanged [49]. CBD exhibits a wide variation in its half-life, with elimination times ranging from 2 to 50 days after oral ingestion [71] and approximately 31 h after inhalation [51]. Cannabinoids are primarily metabolised by enzymes of the CYP family. However, CBD acts as a competitive inhibitor of these enzymes, which may lead to drug interactions with drugs metabolised by the same pathways [13,72]. In fact, studies have reported that this concomitant administration can triple plasma concentrations of active compounds [73]. CBD can also give rise to polymorphisms, which may result in reduced drug efficacy, side effects and interactions [74]. Finally, it was also described that CBD and THC can interact, interfering with their pharmacodynamics [37]. CBD has demonstrated inhibitory properties on drug metabolism [75,76], as well as the metabolic hydroxylation of THC in humans [77]. However, other studies describe the possible potentiation of the effects caused by THC, by CBD [37]. This interactive complexity represents an additional obstacle for combined formulations.

After being metabolised, cannabinoids are excreted for days, with 16% of CBD metabolites being excreted in the urine within 72 h, and a high proportion also being eliminated unchanged in the faeces [23,78]. In general, the elimination of cannabinoids is slow and variable [79]. Therefore, it becomes difficult to estimate the elimination time, which is also conditioned by redistribution from adipose tissue, and consequently, to determine safe and stable dosage intervals.

### 2.4. Regulatory Status and Approved Products for Common Indications

Since 2012, with the approval of cannabis for recreational use in some states of the United States of America, Uruguay and Canada, the debate about its prohibition/authorisation has been growing [16]. In fact, both cannabis and cannabis resin are listed in Schedules I and IV of the 1961 United Nations Single Convention on Narcotic Drugs [80]. However, with regard to its use for medicinal purposes, its use is not prohibited by international law [16]. It is important to understand that the term medicinal cannabis can refer to different forms with different legal implications [81]. Thus, there is cannabis in natura, which refers to any part of any plant of the genus Cannabis, including *C. indica*, *C. sativa* and *C. ruderalis* [81]. There is also cannabis extract, which consists of oil extracted from the plant or derived preparations, or cannabinoids, the compounds present in the cannabis plant [81].

In recent years, more countries have allowed the use of medicinal cannabis, so there has been a shift in policies. Still, there are some reservations due to concerns about dependence and adverse effects [81]. The European Medicines Agency (EMA) and the Food and Drug Administration (FDA) have also resisted approving cannabis-based medicines; however, several European countries and some US states have been changing their regulations, allowing the use of medicinal cannabis [81,82,83]. Thus, in Europe, THC can be used in capsules, cannabis flowers can be used in infusions/decoctions or in the form of vaporisation. Marijuana extract can be used as an oral spray [84]. However, most European countries prohibit the use of cannabis for consumption in its natural state, allowing cannabinoid-based medicines [81]. This is the case with Nabiximols (Sativex^®^), which has gained wide acceptance and is authorised for use in most countries of the European Union. This formulation, based on cannabis plant extract, consists of an oromucosal spray containing THC and CBD [85]. Nabiximols is indicated for the treatment of spasticity associated with multiple sclerosis, particularly after previous treatments have failed. Its use has also been authorised for neuropathic pain associated with multiple sclerosis [81,85,86]. Similarly, Nabilona (Cesamet^®^ or Canemes^®^) has also been used in various European countries. It consists of oral capsules containing a synthetic cannabinoid similar to THC, indicated for the treatment of chemotherapy-induced nausea and vomiting in cancer patients [85,86]. The use of Dronabinol (Marinol^®^ or Syndros^®^) has also been approved in some European countries. This medication consists of an oral solution or capsules containing synthetic THC and is indicated for treating anorexia associated with weight loss in patients with AIDS, and nausea and vomiting associated with chemotherapy for cancer [85,86].

In the US, California was the first state to authorise the use of medicinal cannabis, back in 1996 [81]. Currently, several other states have enacted their own laws regulating the use of medicinal cannabis [87]. However, some states require physicians to have a state registration or to complete a brief medical education program on cannabis as a prerequisite for their registration [88]. The FDA has approved Cesamet^®^, Marinol^®^, and Syndros^®^ for therapeutic use in the United States [81,89,90]. Still, nearly all states limit the conditions under which cannabis can be prescribed, sometimes restricting it to cases where the doctor considers that the treatment outweighs the risks to the patient’s health [91,92].

In other countries, such as Canada, the use of cannabis for medicinal purposes has been permitted since 2014 [81]. In 2016, Health Canada created a Cannabis for Medical Purposes Regulation, under which patients authorised to use cannabis can register and produce a limited quantity of cannabis for their own medicinal purposes, or designate another person for this task [93,94,95]. Nabiximols can also be used in the treatment of spasticity or symptomatic relief of neuropathic pain associated with multiple sclerosis [81]. It can also be administered to patients with advanced cancer who experience moderate to severe pain during treatment [81]. Nabilone is also authorised for severe nausea and vomiting associated with cancer chemotherapy. Finally, dronabinol is also permitted for the treatment of AIDS-related anorexia and severe nausea and vomiting associated with cancer chemotherapy [96].

Raw cannabis is only authorised in a small number of countries, namely Canada, Germany, Israel and the Netherlands, as well as some US states, with magistral preparations of cannabis plants being more accepted [81]. The most widely accepted approach is for doctors to specifically indicate the purposes for which patients can use medicinal marijuana and its formulations. Nevertheless, many countries have been changing their legislation over the years to allow the use of these substances [81].

## 3. Clinical Evidence in Less-Common Disorders

The clinical conditions discussed in this section were identified through a structured literature search conducted in PubMed and Scopus, using the following search strategies: *((cannabinoids) OR (phytocannabinoids)) AND (medical applications)* and *((cannabinoids) OR (cannabidiol) OR (medical cannabis) OR (phytocannabinoids)) AND (rare disorders)*. The search was restricted to publications between 2020 and 2025, and only peer-reviewed articles reporting clinical data were considered eligible for inclusion. Studies were excluded if full-text access was not available, if they reported protocols or ongoing trials without published outcomes, or if they focused on clinical indications that are already well established in clinical practice for cannabinoid-based therapies. These included spasticity associated with multiple sclerosis or spinal cord injury, chemotherapy-induced nausea and vomiting, appetite stimulation in palliative care, chronic pain, Tourette syndrome, severe childhood epilepsies (Dravet and Lennox–Gastaut syndromes), and treatment-resistant glaucoma [6]. Consequently, this section focuses exclusively on less-common, emerging, or off-label clinical indications for which cannabinoids are not yet formally approved, allowing a critical appraisal of both positive and negative clinical evidence in areas where therapeutic need remains largely unmet.

When interpreting the findings discussed throughout this section, several important limitations must be acknowledged. Across literature, sample sizes are frequently modest, substantially limiting statistical power and the reliability of effect estimates. Many investigations are further restricted to specific age groups or to a single sex, thereby reducing the generalizability of findings to broader, more heterogeneous patient populations. Participant cohorts are often clinically heterogeneous, particularly with respect to comorbid neurological or systemic conditions, which complicates the attribution of observed effects to cannabinoid-based interventions alone. Additionally, the routine exclusion of individuals with significant cardiovascular or psychiatric comorbidities, as well as the limited representation of ethnically diverse populations, further constrains external validity and limits the applicability of results to real-world clinical settings.

From a design perspective, several studies lack a control or placebo group, while others rely exclusively on observational methodologies. In paediatric and elderly populations in particular, outcome assessments frequently depend on caregiver or parent-reported measures, increasing susceptibility to subjective bias. Most available studies primarily evaluate short-term or acute effects of cannabinoid administration, leaving uncertainty as to whether repeated or long-term use leads to sustained neurobiological changes or clinically meaningful benefits over time. The frequent absence of objective behavioural, functional, or neurophysiological outcome measures further heightens the risk of bias, as improvements may reflect placebo effects, regression to the mean, expectancy bias, or indirect caregiver relief rather than true therapeutic efficacy.

Additional methodological concerns include potential crossover order effects in within-subject designs, insufficient pharmacokinetic and dose–response data, and reduced statistical power for secondary or exploratory outcomes. Collectively, these limitations underscore the need for larger, well-controlled, longitudinal trials incorporating objective outcome measures, diverse participant populations, and rigorous methodological frameworks to more accurately determine the clinical efficacy and safety profile of cannabinoid-based therapies.

Across multiple clinical trials, cannabinoid treatments were generally well tolerated in diverse patient populations [97,98,99,100,101,102,103,104,105,106,107,108,109,110,111]. Most adverse events reported were mild to moderate in severity, transient, and rarely required dose adjustment or treatment discontinuation [97,98,99,101,102,103,105,106,108,109]. Commonly observed AEs included somnolence, fatigue, decreased appetite, gastrointestinal symptoms (diarrhoea, nausea), dizziness, headache, and dry mouth [98,100,101,102,103,104,108,110,111]. Behavioural changes, transient seizure worsening, or increased anxiety were occasionally reported but were typically dose-dependent and resolved spontaneously or with dose adjustment [99,103,104].

Serious adverse events were rare, with only a few cases possibly related to treatment, such as tonic–clonic seizures requiring hospitalisation or persistent fatigue leading to discontinuation in isolated participants [99,103]. Hepatic enzyme elevations were generally mild, transient, and resolved without intervention, with no recurrent or clinically significant laboratory abnormalities reported [97,100,103,104,105,106]. No clinically relevant changes were observed in hematologic parameters, vital signs, or electrocardiographic measures, and drug–CBD interactions were minimal, even with concomitant medications [97,101,105,106].

The temporal pattern of adverse events suggests most occurred early during the titration phase and diminished with continued treatment [100]. Some studies highlighted advantages of specific formulations, such as transdermal CBD, reducing gastrointestinal side effects and avoiding first-pass hepatic metabolism [99], and ZTL-101 showing rapid resolution of side effects overnight, minimising next-day residual effects [107,111]. Across studies, careful dose titration and monitoring, especially in patients receiving concomitant medications such as valproate or benzodiazepines, helped mitigate adverse events [98,100,105,106].

Overall, cannabinoid formulations demonstrated an acceptable safety and tolerability profile, with most adverse events being mild, reversible, and manageable, supporting their potential use as adjunct therapies in various clinical populations [97,98,99,100,101,102,103,104,105,106,107,108,109,110,111].

### 3.1. Refractory Epilepsies Beyond Dravet and Lennox–Gastaut Syndromes

Refractory epilepsies beyond Dravet and Lennox–Gastaut syndromes comprise a heterogeneous group of conditions characterised by persistent seizures despite adequate trials of multiple antiseizure therapies. This category includes drug-resistant focal epilepsies and several developmental and epileptic encephalopathies, often associated with early onset, cognitive impairment, and significant neuropsychiatric comorbidity. Beyond Dravet and Lennox–Gastaut syndromes, cannabinoid-based therapies have been explored in other forms of refractory epilepsy, with emerging but less robust evidence. However, responses are variable and generally less pronounced than in approved indications. The main characteristics and clinical outcomes of the studies discussed in this section are summarised in Table 1.

The evidence reviewed highlights the broad but heterogeneous clinical impact of cannabinoid-based interventions across refractory epilepsies and complex neurodevelopmental conditions. Across diverse disorders, these studies consistently suggest meaningful benefits in seizure burden, behavioural regulation, mood, sleep, and quality of life, often in populations with severe baseline impairment and extensive prior treatment failure. While antiseizure responses are variable and rarely curative, adjunctive cannabinoid treatment appears to exert multidimensional effects that extend beyond seizure control, potentially reflecting neuromodulatory and homeostatic mechanisms. Overall, the findings support cautious optimism regarding clinical utility in highly refractory populations.

### 3.2. Movement, Neurodegenerative and Sleep-Related Disorders

Movement, neurodegenerative, and sleep-related disorders share pathophysiological features that are directly relevant to the pharmacological mechanisms of drugs currently under investigation, particularly those modulating neurotransmission, neuroinflammation, and neural network stability. Dysregulation of excitatory-inhibitory balance, alterations in basal ganglia and cortico-thalamic circuits, chronic neuroinflammatory processes, and impaired synaptic plasticity are common across these conditions and constitute key therapeutic targets. Drugs under study aim to restore network homeostasis, reduce maladaptive neuroinflammatory signalling, and modulate systems involved in motor control, cognition, and sleep-wake regulation. As such, these disorders provide a biologically plausible framework for evaluating treatments that exert broad neuromodulator effects rather than targeting a single symptom domain or disease entity. Table 2 provides a structured overview of study design, patient populations, cannabinoid formulations, and key efficacy and safety findings across the included trials.

The evidence across movement, neurodegenerative, psychiatric, and sleep-related disorders indicates that cannabinoid-based interventions exert broad but condition-specific effects that extend beyond a single symptom domain. In neurodegenerative and movement disorders, findings primarily support feasibility, safety, and modest benefits on agitation, cognition, spasticity, and caregiver burden, with clinically meaningful effects observed even at very low doses in vulnerable populations. In psychiatric conditions, cannabinoids—particularly CBD—demonstrate more consistent anxiolytic and trauma-related cognitive effects, with emerging evidence of network-level modulation despite variable short-term symptomatic change. In sleep disorders, the data are comparatively robust, showing reproducible improvements in subjective sleep disturbance and sleep continuity, albeit without clear formulation superiority. Collectively, these findings suggest that cannabinoids may act as neuromodulatory agents influencing network stability, emotional regulation, and sleep–wake processes.

### 3.3. Rare and Severe Dermatological Disorders

Rare dermatological disorders are frequently associated with chronic inflammation, impaired barrier function, persistent pain, and pruritus, leading to substantial reductions in quality of life and limited therapeutic options. In this context, cannabinoid-based interventions have attracted increasing attention due to their anti-inflammatory, analgesic, and antipruritic properties. Nevertheless, clinical evidence remains heterogeneous and is largely derived from early-phase studies, small cohorts, or exploratory clinical investigations. For clarity and comparability, the available evidence is synthesised in Table 3, highlighting both therapeutic signals and methodological considerations.

Collectively, available evidence suggests that cannabinoid-based strategies may provide symptomatic benefits in rare and severe dermatological and oral inflammatory disorders, particularly when delivered via topical or local formulations. However, conclusions remain constrained by small sample sizes, heterogeneous study designs, and a reliance on subjective or short-term endpoints. Larger, well-controlled clinical trials with longer follow-up and clearly defined quantitative outcomes are required to establish the true therapeutic value of cannabinoids in these challenging dermatological contexts.

### 3.4. Gastrointestinal and Systemic Inflammatory Disorders

The endocannabinoid system plays a recognised role in gastrointestinal motility, visceral sensation, and immune regulation, providing a biological rationale for the investigation of cannabinoid-based therapies in chronic gastrointestinal disorders. Nevertheless, clinical evidence remains limited and heterogeneous, with outcomes frequently diverging between symptomatic relief and objective measures of disease activity. The paradoxical effects highlight the complexity of cannabinoid actions on gut sensory and motor pathways and raise important considerations regarding long-term clinical implications. A detailed summary of the controlled and observational studies evaluating cannabinoid-based interventions in this domain is presented in Table 4.

### 3.5. Negative or Unsuccessful Clinical Outcomes: Lessons Learned

Despite the growing number of clinical studies investigating cannabinoid-based interventions, several well-designed trials have failed to demonstrate consistent or clinically meaningful efficacy across neurodevelopmental, neurological, psychiatric, and sleep-related conditions. Importantly, these negative or inconclusive outcomes are supported by quantitative data derived from individual clinical studies rather than isolated observations, providing valuable insight into the translational limitations of cannabinoid-based therapies. To facilitate cross-study comparison, Table 5 consolidates the principal clinical endpoints, dosing strategies, and safety outcomes reported in the literature.

Taken together, evidence from multiple independent clinical studies consistently demonstrates a lack of statistically significant superiority over placebo across several indications, alongside high placebo responsiveness, reliance on subjective endpoints, heterogeneous patient populations, and, in some cases, exposure-related adverse cognitive effects. These findings emphasise that biological plausibility and widespread use do not guarantee clinical efficacy and underscore the need for adequately powered, indication-specific trials employing robust and objective outcome measures to define the realistic therapeutic boundaries of cannabinoid-based interventions.

## 4. Formulation Strategies, Safety Considerations and Regulatory Aspects

### 4.1. Why Formulation Matters in Rare Disorders

Cannabinoids have received increasing attention for their therapeutic potential, although their pharmacokinetics are not fully understood [141]. Regardless of the route, once absorbed, cannabinoids are rapidly distributed systemically [142]. However, only 5% of CBD and THC do not bind to plasma proteins and are therefore responsible for the pharmacological effect [143]. Thus, one of the biggest determinants of the bioavailability of these compounds is related to the form of administration and, above all, to the formulation [142].

Cannabinoids have the ability to inhibit cytochrome P450 enzymes, namely CYP2C9 and CYP34A, which is why potential drug interactions may occur [142]. In fact, inhibiting these enzymes can alter the concentration of drugs in the plasma, leading to an increase in their concentration, which may result in toxicity or more adverse effects [144]. Additionally, given the inhibition of cytochrome P450 enzymes, the combined administration of THC and CBD may result in significant changes in the metabolism of these compounds [12].

The effects of cannabinoids are highly influenced by inter-individual variability, since factors such as genetics, physiology, and environment can affect therapy with these compounds. According to Wright et al. [145], there is a wide spectrum of potential changes in THC and CBD metabolism that contributes to pronounced interindividual variability in response. The results suggest that individuals classified as slow, normal, and ultra-rapid metabolizers for CYP2C9 and CYP34A may exhibit substantial differences in how they process THC and CBD, which could lead to divergent therapeutic outcomes. At the same time, changes in liver function or transporter activity can affect the pharmacokinetics of cannabinoids, influencing both efficacy and tolerability and further increasing interindividual variability [146]. One of the administration routes most influenced by interindividual variability is the oromucosal route. Factors such as differences in saliva production, absorption by the oral mucosa, and swallowing patterns significantly affect the absorption of cannabinoids, thus influencing therapeutic outcomes [147].

A study developed by Reddy et al. [148], demonstrated that pharmacokinetics can be improved by altering the formulations and their excipients. Strategies to improve the pharmacokinetics of cannabinoids focus on overcoming limitations such as low water solubility, first-pass metabolism and variability in absorption [148]. To overcome these limitations, approaches such as the use of lipid-based formulations or emulsions, which increase solubility and facilitate intestinal absorption, are important. Another approach worth mentioning is encapsulation in micro or nanoemulsions and lipid capsules, which protect the molecule and improve systemic delivery. Thus, to avoid first-pass metabolism, the use of transdermal and intranasal routes may constitute a viable alternative [149].

Another important factor for the effectiveness of cannabinoid treatments is patient adherence. Like all medications, both CBD and THC are associated with adverse effects, which can compromise adherence to and compliance with treatment. According to Pomey et al. [150], patients discontinue cannabinoid-based therapies mainly due to limited efficacy and adverse effects. In the case of rare diseases, the very heterogeneity of patients creates a significant obstacle. Furthermore, the small number of people affected makes the process even more challenging [151]. Similarly, the use of orphan drugs is also significant in the treatment of rare diseases. The choice and development of the formulation of these medications are fundamental to ensuring effective and appropriate results in the different profiles of patients with rare diseases [152].

### 4.2. Relevant Pharmaceutical Approaches

#### 4.2.1. Oromucosal Sprays

Historically, oral administration of medication was the most common and accepted approach, due to its convenience and non-invasive nature. Initially intended for local effects, sublingual and buccal administration began to be used for systemic administration. This last one allows for faster action and better patient adherence, being considered a good alternative to intravenous administration [153]. The oral cavity is the first part of the gastrointestinal tract, extending from the mouth to the beginning of the pharynx, and is made up of the buccal, sublingual, gingival, palatine and labial mucosa [154]. The oral mucosa is characterised by being composed of non-keratinised tissue, which makes it more permeable and elastic [155]. Another important characteristic of this epithelium is that, although rigid, small molecules can pass through it, potentially avoiding first-pass metabolism. However, it is important to note that drug absorption may be limited due to the small contact area and the processes of swallowing and saliva production [155]. Therefore, for medications to be absorbed through the oral mucosa, they must first be dissolved in saliva, the volume of which is significantly lower in the mouth. On the other hand, high saliva concentration can lead to premature swallowing, resulting in inadequate drug release [154].

The drug must then diffuse through the mucosa itself, which is determined by lipophilicity and the degree of ionisation. To diffuse through the mucosa, drugs can permeate via both transcellular and paracellular pathways. Most lipophilic molecules diffuse via the transcellular pathway, while hydrophilic molecules permeate via the paracellular pathway [155]. In order to overcome these limitations, alternative administration methods have been developed, primarily through mucoadhesion and the use of mucoadhesive polymers.

Another strategy that has become very relevant is the use of medications with rapid disintegration of the drug and consequent almost immediate release, as is the case with sprays [154]. Oromucosal sprays are liquid formulations applied directly to the oral mucosa, allowing medications to be absorbed by the oral epithelium for local and systemic effects. Compared to conventional oral administration, they offer greater bioavailability, avoiding first-pass metabolism, and providing a faster onset of action due to direct systemic absorption. The main advantage is greater convenience for patients, especially those with swallowing difficulties, such as children, the elderly and uncooperative patients, as is the case with some patients with rare diseases [154,156].

Nabiximols are botanical preparations containing balanced amounts of THC and CBD and have been used as an oromucosal spray (Sativex^®^) for patients with multiple sclerosis with moderate to severe spasticity [157]. Studies have shown that nabiximols has significant efficacy in treating the symptoms of multiple sclerosis, showing that this is a consistent therapy, even as monotherapy [157]. Nabiximols have also been used in the treatment of Tourette syndrome [158]. Müller-Vahl et al. [158] carried out a study with nabiximols, where they found a greater number of responders compared to the placebo group. However, the difference was not statistically significant. Secondary analyses indicated that patients with Attention Deficit Hyperactivity Disorder (ADHD) showed a decrease in severe tics. Thus, the study showed that nabiximols may be a good approach to reduce tics in Tourette syndrome [158].

#### 4.2.2. Transdermal/Topical: Gels and Patches

The skin is the largest organ in the body, composed of five layers, including an outer layer, the *stratum corneum*, which acts as a barrier against hydrophilic substances and large molecules [159]. Transdermal drug delivery systems (TDDS) and topical formulations are a promising non-invasive method for delivering active drugs across the skin barrier [160]. Typically, topical drug administration refers to the treatment of a localised area of skin, while TDDS refers to the administration of drugs through the skin and into the systemic circulation [161]. TDDS tablets are composed of several layers that facilitate the absorption of the medication. The support layer acts as an external protective barrier, shielding the system from the external environment. Next, the adhesive layer attaches the patch to the skin using a hypoallergenic adhesive that is gentle on the skin. At the core, the drug reservoir contains the active pharmaceutical ingredient, which is released at a constant rate through a membrane [159]. On the other hand, gels are systems formed by a polymer and a solvent, arranged in a three-dimensional structure in a cross-linked polymer network and have different drug delivery systems [162].

Drug penetration through the skin requires passage through both the stratum corneum and the skin’s cellular matrix. Drug penetration into the skin occurs through transcellular permeation and intercellular absorption. Transcellular permeation involves the direct absorption of drugs through individual skin cells, while intercellular absorption occurs within the extracellular matrix through the interstitial spaces between neighbouring cells. Another way drugs are absorbed through the skin is through skin appendages, such as hair follicles and sebaceous glands [161].

When developing effective drug delivery systems, several variables must be considered, namely, active pharmaceutical ingredients and skin morphology [161]. Most active pharmaceutical ingredients do not inherently meet the criteria for effective transdermal administration, so it is important to develop new strategies to improve their absorption [160]. Thus, organogels have been used in transdermal delivery systems to improve the transdermal administration of hydrophilic and hydrophobic drugs that present lipophilicity problems [159]. In recent years, several nanocarrier formulations have also been developed to improve transdermal drug delivery, including liposomes and polymeric micelles [163].

Cannabinoids are known for their medicinal properties, especially as anti-inflammatories. Its topical application as anti-inflammatory compounds has been at the forefront of research in the last decade, also receiving increasing attention in the cosmetics field, as it can help alleviate skin problems due to its topical anti-inflammatory effect [163]. However, unlike transdermal delivery systems, such as cannabinoid patches, this route does not involve systemic absorption [164]. Thus, transdermal delivery systems have gained great relevance not only because of the possibility of systemic absorption, but also because they help to bypass first-pass metabolism, increasing user adherence [165]. Studies have shown that emerging transdermal systems, such as transdermal patches, can significantly increase CBD absorption and therefore help in the treatment of skin conditions such as dermatitis and even epidermolysis bullosa, due to their anti-inflammatory action [166]. An experimental topical cream, INM-755, was tested for the treatment of epidermolysis bullosa [167]. In phase II studies, this topical formulation demonstrated good tolerability and safety, without negatively interfering with the healing process. Therefore, the absence of serious adverse effects on such fragile skin and the good acceptance by participants indicate that this type of formulation is suitable for repeated cutaneous application [167]. Another study with the transdermal gel ZYN2-CL-017, which contains CBD, investigated long-term efficacy and safety in populations with fragile X syndrome [128]. The main results of the study show a favourable safety profile and revealed clinically significant improvements [128]. Furthermore, these studies support the idea that transdermal formulations can be effective vehicles for the local and systemic administration of cannabinoids in rare diseases.

Despite some promising results, these systems still have inherent limitations, such as skin permeability, which can be overcome with permeability enhancers like ethanol and oleic acid. Furthermore, a preclinical study with guinea pigs demonstrated that the addition of transcutol HP, a permeation enhancer, increased plasma CBD concentration by 3.7 times when added to a topical CBD gel [168].

Physical permeation enhancers, such as microneedles, can solve the problem of cannabinoid permeation, but studies in this area are still few [164].

#### 4.2.3. Nanocarriers

As previously described, cannabinoids have lower solubility and are easily subjected to oxidation and degradation reactions due to the action of light and temperature. These limitations make them interesting candidates for nanotechnology-based formulations [169]. The technology of encapsulating cannabinoids in nanocarriers has become a good bet to protect the compounds from degradation, increasing their stability [148]. In this regard, both lipid-based carriers and polymeric carriers have been investigated regarding their mode of action.

Polymeric nanocarriers can be produced in capsules and spherical shapes, allowing for better release, while lipid nanocarriers have been shown to favour targeted delivery [170]. Among lipid-based nanocarriers, nanoemulsions showed increased CBD absorption, demonstrating that bioavailability can increase up to 1.65 times, significantly reducing the time to reach peak plasma concentration. However, due to high production costs and instability, Self-Nanoemulsifying Drug Delivery Systems (SNEDDS) emerged, which consist of self-emulsifying systems that spontaneously form nanoemulsions in the gastrointestinal tract [171]. Evidence shows that these not only increase the solubility and stability of cannabinoids, but also their bioavailability. However, most studies consist of small clinical trials, so larger clinical trials are still needed [148]. Additionally, despite being a promising alternative, SNEDDS do not avoid the first-pass mechanism [171].

Liposomes, on the other hand, are spherical vesicles made up of phospholipids and cholesterol, in which one or more layers of phospholipids surround an aqueous core. Although these systems are widely studied, they have low encapsulation efficiency for cannabinoids. Even so, studies in dogs with osteoarthritis showed CBD bioavailability 17 times greater than that of free CBD, demonstrating that encapsulation increases CBD activity, even at reduced doses [169]. On the other hand, there are also polymeric micelles, which consist of amphipathic nanoparticles with a hydrophobic core and a hydrophilic layer, used as reservoirs for lipophilic drugs, such as cannabinoids. Studies have shown that polymeric nanoparticles allow for greater bioavailability. Studies with Polylactic-co-Glycolic Acid (PLGA) nanoparticles loaded with CBD showed rapid initial release and high encapsulation efficiency [171]. Villate et al. [172] developed a study with PLGA nanocapsules loaded with full-spectrum cannabis extract, demonstrating that these formulations protect cannabinoids from gastric degradation and allow their controlled release in the intestine, increasing the local concentration of cannabinoids. Thus, the study demonstrated that, with biocompatible polymers, nanotechnology can be promising in the treatment of gastrointestinal diseases [172]. However, most clinical evidence remains in vitro or in vivo models, so clinical validation is still limited [171].

### 4.3. Safety Profile Across Rare Conditions

Recreational use of cannabinoids is associated with very worrying side effects, namely psychosis, schizophrenia and cannabis use disorder, especially in adolescents [173]. The adverse effects associated with the use of cannabinoids for medicinal purposes are linked to an increased risk of short-term side effects but are rarely associated with serious effects. In fact, products containing medicinal THC are often associated with changes in perception and thinking, as well as dizziness and sedation, particularly in the elderly. However, CBD does not cause intoxication and presents fewer safety concerns than THC. Still, potential side effects, such as liver toxicity and drug interactions, as well as inadequate regulatory oversight of CBD products, may constitute legitimate concerns [5].

The main adverse effects of prolonged use of cannabinoids include gastrointestinal side effects, namely vomiting, cardiovascular effects such as tachycardia and orthostatic hypotension, and, mainly at the psychiatric level, an increased risk of depression and suicidal ideation [142]. Furthermore, CBD has been reported to cause liver abnormalities, diarrhoea, fatigue and drowsiness in some individuals [141]. Another problem that has been reported is the potential for interaction with other medications. According to a recent study developed by Nachnani et al. [174], cannabinoids can significantly alter the action of many medications, especially those with a narrow therapeutic index. The study reports that cannabinoids interact with warfarin, increasing its clotting time [174]. Other medications, such as tricyclic antidepressants and anticonvulsants like valproate, have also shown significant interactions with CBD [174]. Another study showed that CBD is the main culprit behind interactions with other medications. CBD primarily inhibits CYP2C19, CYP2C9, CYP3A and CYP1A2; therefore, interactions occur mainly during first-pass metabolism [175]. These results indicate that CBD increases exposure to the drug by inhibiting its initial clearance [175]. In fact, the medications with the highest risk of interaction with CBD are those that are metabolised by the enzymes mentioned above. Thus, antidepressants, opioids, benzodiazepines, antihypertensives and anticonvulsants have significant interactions because they are extensively metabolised by cytochrome P450 family enzymes [176]. Epidiolex^®^ is approved for refractory epilepsies, including rare diseases such as Dravet and Lennox–Gastaut [177]. Despite its favourable safety profile, it can cause pharmacokinetic changes and interactions with other anticonvulsants. Concomitant administration with clobazam increases levels of its metabolite, increasing the risk of sedation [177]. Changes in liver enzyme levels can also occur with valproate. Minor interactions were observed with topiramate and levetiracetam; therefore, dose adjustments may be necessary [177].

Another legitimate concern when using medicinal cannabis is its administration to children. In fact, most studies on cannabinoids are conducted in adult animal models; therefore, research on long-term adverse effects in children and adolescents is still limited. As a result, there is some uncertainty about how cannabinoids affect a developing brain [178]. Children are very vulnerable to cannabinoid treatments because their pharmacokinetics vary greatly due to the immaturity of their physiological system. Consequently, oral absorption is less effective, and distribution is affected by the low percentage of fat. Metabolism is also affected by liver enzymes, which are still developing. Therefore, children are equally susceptible to drug interactions. In the specific case of concomitant use with antiepileptic drugs, it should be noted, once again, that CBD significantly increases the concentration of clobazam. It is also important to report the interaction of antidepressants such as sertraline with CBD in children, which can be equally dangerous [179].

Other vulnerable groups, particularly transplant patients whose immune systems are suppressed, have also been a cause for concern. According to a review on the use of CBD in post-organ transplant care, the use of cannabis has been shown to be a good supportive therapy for the relief of chronic pain [180]. However, this group is equally susceptible to the use of cannabinoids, as they can interfere with immunosuppressant medications. Studies report that CBD may interfere with the concentration of tacrolimus and other immunosuppressants, increasing their blood concentration and potentially resulting in increased toxic effects [142,181,182,183,184]. Thus, the use of cannabinoids should also be rigorously monitored in immunosuppressed patients [180].

### 4.4. Regulatory Considerations

With the expansion of the regulatory framework and the market for cannabis-derived products, the variety of cannabinoid products has increased significantly for both recreational and medicinal use, including in the treatment of diseases for which this use is not indicated. Thus, when these products are used without solid regulatory support regarding safety, patients may be exposed to uncertain risks [185].

The FDA acknowledges that there is growing interest in the therapeutic potential of cannabis in treating diseases, but so far has not approved any marketing authorisation applications for its use. The only approved medicines are cannabis-based, namely Epidiolex^®^ and three synthetic cannabis-based medicines, such as dronabinol (Marinol^®^ and Syndros^®^) and nabilone (Cesamet^®^) [83]. With regard to Europe, only dronabinol, nabilone and, in particular, nabiximols have been authorised by the EMA in European member countries. Nevertheless, the regulation of compounded preparations is a national responsibility, leading some Member States to independently authorise the prescription and sale of cannabinoid products [186].

Thus, medicinal cannabis and related products have been available in the Member States of the European Union as individual prescriptions without regular marketing authorisations [187]. Therefore, there is no specific framework for cannabis-based medicines in Europe. Depending on their composition, they may be considered medicinal plant-based products, which can be authorised for the market through registration for traditional use. In the US, despite federal regulations, state laws vary, allowing, in some cases, the medicinal use of cannabis without FDA approval [188]. However, there are very few approved cannabis-based medicines, so their use beyond the indicated options is considered off-label [188].

The FDA acknowledges that there are cannabinoid-based drugs being used for unofficial purposes, highlighting the importance of approved drugs undergoing rigorous evaluations, unlike unapproved products, which can cause unpredictable and serious adverse effects, since there are no clinical trials to prove their safety [83]. Thus, only standardised pharmaceutical cannabinoids are approved by the EMA and FDA, such as Epidiolex^®^, dronabinol, nabilone and nabiximols (Sativex^®^), while the use of other medicinal cannabis products remains outside the regulatory scope, without quality assurance and, especially, safety [187,188].

Epidiolex^®^ is approved by the EMA and the FDA for the treatment of seizures associated with Lennox–Gastaut syndrome, Dravet syndrome or tuberous sclerosis complex in patients 1 year of age or older, especially with orphan drug designation: a drug used for the diagnosis and treatment of rare diseases [189,190].

Generally, orphan drugs are supported by incentives such as tax breaks and market exclusivity to encourage their development, despite serving a small patient population [191]. According to Orphanet, a major database of orphan drugs, they are intended to treat diseases so rare that the market is reluctant to develop them under normal market conditions, given their expensive and time-consuming development, which makes rare diseases unattractive to the pharmaceutical industry [152,192]. It is therefore understandable that these medicines are subject to certain limitations, such as high research and development costs, small patient populations involved and the regulatory and market dynamics that govern the industry [191]. To overcome market limitations, in particular, the Orphan Drugs Act (1983) [152] in the United States granted seven years of market exclusivity with tax exemptions to encourage the development of orphan drugs. Meanwhile, in Europe, the Orphan Medicinal Products Regulation (2000/2001) grants ten years of exclusivity with the same fee exemptions. It should be noted that both the FDA and the EMA assist in clinical trials for small populations [152].

Rare diseases also involve very small patient populations, which makes recruitment difficult and often renders traditional clinical trials unfeasible. To overcome this limitation, innovative methods, such as master protocols, have been used, but even so, they require ethical considerations and informed consent, which makes the process more time-consuming, limits the number of volunteers and, consequently, the existence of more robust studies [191]. The approval of orphan drugs, as often happens in smaller studies, can lead to the approval of therapies with uncertain safety and efficacy profiles. Therefore, more robust studies are important to detect risks that may not be evident in smaller studies [193,194]. Furthermore, post-marketing pharmacovigilance is essential to identify safety signals that do not appear in clinical trials or that are specific to subgroups, namely, in patients with rare diseases [195].

## 5. Conclusions

Cannabinoid-based therapies are increasingly recognised as pharmacologically plausible interventions beyond their currently approved indications. The evidence reviewed in this article highlights a growing, yet heterogeneous, body of clinical data supporting the potential role of cannabinoids in less-common, rare, and complex disorders, particularly in conditions characterised by high symptom burden and limited therapeutic alternatives. Across neurological, neurodevelopmental, psychiatric, dermatological, gastrointestinal, and sleep-related disorders, cannabinoids, most notably CBD, have demonstrated multidimensional effects extending beyond single symptom domains, including modulation of seizure burden, behaviour, mood, sleep, pain, and quality of life. Thus, cannabinoids may play an important role in the development of innovative therapies, particularly in the treatment of less-common diseases that often lack effective therapeutic options.

Importantly, the reviewed studies illustrate that clinical responses to cannabinoids are highly variable and context dependent. While some patient populations experience clinically meaningful benefits, others derive limited or no measurable advantage, as exemplified by well-conducted trials reporting neutral or negative outcomes. This highlights the need for more targeted studies in specific populations and clinical conditions. Recent studies suggest that specific effects in the brain region, which affect behavior, may be closely related to cannabinoid compounds, leading to the belief that this class of compounds presents a relevant (poly)pharmacology. Other findings underscore the necessity of avoiding overly generalised conclusions regarding cannabinoid efficacy and instead adopting a nuanced, indication-specific interpretation of available evidence. The inclusion of negative and inconclusive trials is particularly instructive, highlighting methodological challenges, placebo effects, and the limitations of current outcome measures. Therefore, the development of larger and better-controlled clinical trials is essential.

Pharmaceutical formulation emerges as a critical determinant of therapeutic success. Poor aqueous solubility, variable bioavailability, extensive first-pass metabolism, and inter-individual pharmacokinetic variability remain major obstacles to consistent clinical outcomes. Advances in drug delivery systems, including oromucosal, transdermal, and nanocarrier-based formulations, offer promising strategies to mitigate these limitations and may partly explain discrepancies observed across clinical studies. Optimising formulation and route of administration is therefore not merely a technical consideration but a central component of effective cannabinoid-based therapy, especially in vulnerable and rare disease populations.

From a safety perspective, cannabinoids generally exhibit acceptable tolerability profiles when appropriately dosed and monitored. Nevertheless, clinically relevant drug–drug interactions, particularly involving cytochrome P450 enzymes, and dose-dependent adverse effects reinforce the need for careful patient selection, titration, and longitudinal surveillance. Regulatory heterogeneity across jurisdictions further complicates clinical translation, reflecting ongoing uncertainties regarding risk–benefit balance and evidentiary thresholds.

## Figures and Tables

**Table 1:** Cannabinoids in Refractory Neurological and Neurodevelopmental Disorders Beyond Dravet and Lennox–Gastaut Syndromes.

Ref.	[97]	[112]	[101]	[104]	[102]		[113]
**Safety**	Reported as safe and well tolerated (no major safety signals described)	Not specifically detailed	No major safety concerns reported	Well tolerated; no significant neuroscore changes	Somnolence correlated with response; generally acceptable tolerability		Suggests comparatively good tolerability
**Key Findings**	Median seizure reduction 82%; 4/5 achieved ≥50% reduction; 3/5 > 75%; median seizures reduced from 32/month to ~6–7/month; 4 patients reduced/discontinued ≥1 ASM	Parental-reported improvements in irritability, emotional regulation, social responsiveness; 22 lipid biomarkers identified (46% significantly modulated); modulation of sphingolipids/phospholipids/fatty acids suggesting homeostatic effect	90% improved in ≥1 severe symptom; 83.5% of symptoms improved; 30–40% mean improvement in irritability, withdrawal, hyperactivity; 50% improved RRBs; modest sleep improvement	Significant seizure reduction at week 14 and follow-up; 3/5 sustained ≥50% reduction; improved QoL; subjective functional gains	49% “much/very much improved” with whole-plant vs. 21% placebo; modest BMI reduction; male sex/younger age associated with greater response; dose-response trend		9/34 received CBD; partial seizure reduction in majority; no sustained seizure freedom; lower withdrawal rate vs. other ASMs
**Main** **Outcomes**	Seizure frequency reduction	Behavioural domains; salivary lipid biomarkers	Behavioural scales (ABC, Vineland-II), sleep, parental stress	Seizure frequency; quality of life; neuroscore	Clinical Global Impression–Improvement; behavioural scales; BMI		Seizure control; long-term outcomes
**Compound/Formulation** **Dose & Duration**	CBDV 2.5 mg/kg/day titrated to ~10 mg/kg/day; duration not explicitly stated	Individualised medical cannabis treatment (CBD 7.5–200 mg/dose; THC 0.05–50 mg/dose; up to TID) ≥1 year	Purified CBD Median initial dose 138.75 mg; median total 363.5 mg; median follow-up 11 months	Adjunctive CBD 5–25 mg/kg/day; long-term extension up to 63–80 weeks	Whole-plant CBD:THC (20:1); purified CBD:THC (20:1) 12 weeks		Oil-diluted cannabis extract, Bedrocan^®^ 22% THC, 0.5% CBD, Olive Oil 50 mL, twice a day for 12 weeks
**Population**	5 female children; median age 12.6 years; severe drug-resistant epilepsy	15 children (mean age 9.4 years) on medical cannabis ≥ 1 year; 9 controls	20 paediatric patients (85% male); mean age 10 ± 4.6 years; multiple psychotropics	5 patients (4F, 1M); mean age 8.8 ± 6.3 years	150 participants aged 5–21 years		34 patients (30F, 4M); age 1–28 years; early-onset refractory epilepsy
**Study Design**	Phase 1, open-label	Observational	Prospective, observational, before–after	Open-label exploratory study	Randomised, double-blind, placebo-controlled trial		Multicentre observational
**Disease**	Rett Syndrome (MECP2-related) with refractory epilepsy	Autism Spectrum Disorder	Autism Spectrum Disorder with intellectual disability	Sturge–Weber Syndrome with treatment-resistant epilepsy	Autism Spectrum Disorder (severe)		CDKL5 Deficiency Disorder
**Ref.**	**[103]**	**[100]**	**[114]**	**[98]**		**[99]**	
**Safety**	No cognitive deterioration; well tolerated	Dose-dependent transaminase elevations, especially with valproate	No major safety concerns reported (acute study)	Generally well tolerated; no major safety signals highlighted		Acceptable tolerability; no major safety concerns	
**Key Findings**	Significant improvement in neurological function and QoL; reduced anxiety, depression, emotional dysregulation; stable cognition	Early efficacy (Day 6–10); median seizure reduction 37% (CBD25) & 36% (CBD50) vs. 18% placebo; higher ≥50% responder rates	CBDV modulated atypical striatal connectivity; attenuation of hyperconnectivity in language/social circuits; mechanistic relevance to ASD domains	86% responders; 95.4% with baseline depression improved; mood/anxiety improvements independent of seizure response; QoL improved in 68%		No significant effect during blinded phase; 60.8% achieved ≥50% reduction by month 6 in OLE; sustained reductions in long-term follow-up	
**Main** **Outcomes**	Neuroscore; QoL; behavioural and anxiety scales	Seizure frequency; timing of efficacy; AEs	Resting-state fMRI connectivity	Seizure response; depression (BDI-II); anxiety; QoL (QOLIE-10)		Seizure frequency	
**Compound/Formulation** **Dose & Duration**	Oral CBD 5–20 mg/kg/day for 6 months	Purified plant-derived CBD 25 or 50 mg/kg/day; 16 weeks	CBDV acute administration Single-dose 600 mg	Highly purified CBD oil 250 mg/day, dose adjustment, escalation by 1 mL (100 mg), reaching 500 mg/day 6 months, adjustment every 4 weeks		Transdermal CBD (195 mg or 390 mg) 12-week RCT + OLE up to 2 years	
**Population**	10 patients (6F,4M); mean age 13.8 ± 9.7 years	224 patients aged 1.1–56.8 years	28 adult men (13 cases, 15 controls)	44 adults		150 participants (age 5–21 years)	
**Study Design**	Prospective, open-label pilot	Post hoc analysis of Phase 3 RCT (double-blind, placebo-controlled)	Double-blind, placebo-controlled, repeated-measures pilot	Prospective, observational, open-label cohort		Randomised, double-blind, placebo-controlled; open-label extension	
**Disease**	Sturge–Weber Syndrome (controlled seizures)	Tuberous Sclerosis Complex–related drug-resistant epilepsy	Autism Spectrum Disorder	Drug-resistant focal epilepsy		Drug-resistant focal epilepsy	

**Table 2:** Cannabinoids in Movement, Neurodegenerative, Psychiatric, and Sleep Disorders.

Ref.	[115]	[106]	[116]	[105]		[117]			[118]
**Safety**	Well tolerated; no intervention-attributable serious Adverse Effects	Sub-milligram dosing; no major safety concerns reported	Generally well tolerated; uncontrolled design limits inference	Increased drowsiness vs. placebo		Mild Adverse Effects (somnolence, nausea); overall favourable tolerability			No major safety concerns reported
**Key Findings**	No substantial benefit for agitation; demonstrated feasibility in the frail elderly population	Significant MMSE advantage vs. placebo; 64% maintained/improved vs. 33% placebo; relative cognitive stabilisation	Reduced agitation, irritability, apathy, sleep disturbance; decreased caregiver distress; heterogeneous cognitive effects	No significant efficacy differences vs. placebo; subjective improvements in relaxation, communication, sleep in subset		No significant symptom or cognitive change; plasma THC increased more in the placebo group			CBD reduced anxiety and cognitive impairment in the nonsexual trauma subgroup; no effect in the sexual trauma subgroup
**Main** **Outcomes**	Agitation	MMSE cognitive performance	NPI-Q; CMAI; MMSE	Spasticity; caregiver-reported outcomes		Symptom severity; cognition			Subjective anxiety; cognitive impairment
**Compound/Formulation** **Dose & Duration**	Nabiximols (THC:CBD 1:1 oromucosal spray) 8 weeks (4-week titration + 4-week treatment)	Balanced THC–CBD oral extract (THC 0.350 mg + CBD 0.245 mg daily) 26 weeks	THC-dominant extract (Bedrocan^®^; ~22% THC, 0.5% CBD) Twice daily for 12 weeks	Full-spectrum cannabis oil (CBD:THC 10:1) 6-week double-blind phase + 6-week open-label extension phase		CBD 600 mg/day 28 days			Single oral CBD 300 mg Acute administration prior to trauma recall
**Population**	29 nursing home residents (moderate–severe cases)	29 patients aged 60–80 years	30 patients aged 65–90 years	53 patients aged 5–25 years		31 clinically stable individuals (≤5 years diagnosis)			33 adults
**Study Design**	Randomised, double-blind, placebo-controlled feasibility trial	Phase II randomised, double-blind, placebo-controlled trial	Retrospective observational case series	Prospective, double-blind, randomised, placebo-controlled trial		Randomised, double-blind, placebo-controlled add-on trial			Randomised, double-blind, placebo-controlled experimental study
**Disease**	Alzheimer’s disease–related dementia (agitation)	Alzheimer’s disease–associated dementia	Alzheimers disease	Severe spastic cerebral palsy (Gross Motor Function Classification System IV–V)		Psychotic disorders (recent onset, cannabis users)			Post-traumatic Stress Disorder
**Ref.**	**[119]**	**[120]**	**[121]**	**[107]**	**[109]**		**[111]**	**[108]**	
**Safety**	Well tolerated	Acute administration; no major safety issues described	Well tolerated; no serious Adverse Effects	Short-term use well tolerated	Well tolerated; no cognitive impairment		Well tolerated	No major safety concerns reported	
**Key Findings**	Reduced recall-induced cognitive impairment; effect persisted at 1 week; limited anxiolytic effect at 300 mg	Increased vmPFC activation in PTSD (suggesting partial normalisation of extinction circuitry); increased amygdala activation during renewal; no behavioural change	Mean GAD-7 reduction −7.02 vs. placebo; HAM-A −11.9; significant improvements in anxiety, depression, sleep	Significant ISI reduction; ↓ sleep onset latency; ↑ total sleep time (>1 h); ↑ sleep efficiency; large effect size	No major ISI change; ↑ sleep efficiency; transient sleep quality improvement; improved well-being		No cognitive deterioration; improved calmness, alertness, energy; possible mood benefits	~2/3 achieved clinically meaningful improvement; no overall superiority between formulations	
**Main** **Outcomes**	Cognitive impairment after recall	Neural activation (vmPFC, amygdala); fear extinction	GAD-7; HAM-A; CGI; PHQ-9; PSQI	ISI; sleep diary; actigraphy	Sleep efficiency (actigraphy); sleep quality; WHO-5		Neurocognition (CogPro); mood states	PROMIS Sleep Disturbance	
**Compound/Formulation** **Dose & Durationtion**	Single oral CBD 300 mg Acute + 1-week follow-up	Single administration prior to conditioning/extinction task Acute low-dose oral THC	Nanodispersible oral CBD solution (150 mg/mL) 15 weeks	ZTL-101 sublingual cannabinoid extract Two 2-week treatment periods	Sublingual CBD 150 mg nightly 2 weeks		Sublingual CBD 150 mg nightly 2 weeks	CBD isolate 15 mg; CBD + CBN ± CBC; melatonin ± cannabinoids 4 weeks	
**Population**	33 adults	71 participants (19 cases; 26 TEC; 26 HC)	178 adults (89 CBD; 89 placebo)	23 adults; mean age ~53 years	30 adults (15 CBD; 15 placebo)		30 adults (15 CBD; 15 placebo)	1298 adults; mean age ~46 years (mixed population)	
**Study Design**	Randomised, double-blind, placebo-controlled experimental study	Randomised, double-blind, placebo-controlled fMRI study	Phase III multicentre randomised, double-blind, placebo-controlled trial	Randomised, double-blind, placebo-controlled crossover (Phase 1b)	Randomised, placebo-controlled pilot (parallel)		Randomised, double-blind, placebo-controlled	Large randomised, double-blind comparative effectiveness trial	
**Disease**	Post-traumatic Stress Disorder (memory reconsolidation focus)	Post-traumatic Stress Disorder (fear extinction paradigm)	Mild–moderate anxiety	Chronic insomnia	Primary insomnia		Primary insomnia (daytime cognition study)	Sleep disturbance	

Caption: ↑ increase; ↓ decrease.

**Table 3:** Cannabinoids in Rare and Severe Dermatological and Oral Inflammatory Disorders.

Ref.	[122]	[123]	[124]
**Safety**	Not yet reported (trial ongoing)	Well tolerated; no serious Adverse Effects	No safety concerns reported
**Key Findings**	Trial initiated to address prior anecdotal evidence; efficacy results pending; methodological advancement with quantitative endpoints	Sustained pruritus reduction; improved clinical severity scores; improved epidermal barrier function; reduced corticosteroid requirement (steroid-sparing effect)	Significant reductions in gingival index and bleeding vs. placebo; supports the feasibility of local cannabinoid delivery
**Main Outcomes**	Affective pain (validated pain scales); overall pain; pruritus; rescue analgesic use; functional neuroimaging	Pruritus severity; eczema area and severity indices; transepidermal water loss; corticosteroid use	Gingival index; bleeding on probing
**Compound/Formulation** **Dose & Duration**	CBM oil/THC (100 mg/mL)/CBD (50 mg/mL) (Transvamix^®^); 1 mL sublingually administration; Maximum Dose: 0.75 mL/day, 4 administrations/day;. 64 days (baseline measurements, two intervention phases, washout period, follow-up)	Topical oil-in-water emulsion containing CBD and ginger extract (lipophilic CO_2_ extract) Topical application for 12 weeks	CBD-containing toothpaste and dental gel 56 days (adjunct to oral hygiene)
**Population**	28 Adults (≥16)	100 Adult and paediatric patients	90 Adults
**Study Design**	Randomised, double-blind, placebo-controlled crossover trial (ongoing)	Clinical study	Randomised, double-blind, placebo-controlled trial
**Disease**	Epidermolysis bullosa (chronic pain)	Atopic dermatitis (mild–moderate; refractory cases included)	Periodontitis (gingival inflammation)

**Table 4:** Cannabinoids in Gastrointestinal and Systemic Inflammatory Disorders.

Ref.	[125]	[126]
**Safety**	No major safety concerns reported in text	No major safety concerns reported; physiological slowing of gastric emptying noted
**Key Findings**	Significant reduction in Crohn’s Disease Activity Index (median 282→166; *p* < 0.05) and improved QoL vs. placebo; no significant changes in endoscopic scores or inflammatory biomarkers; no evidence of mucosal healing	Significant reduction in total Gastroparesis Cardinal Symptom Index (*p* = 0.008); improved nausea, vomiting, meal completion; increased tolerated intake volumes; paradoxical slowing of gastric emptying
**Main Outcomes**	Crohn’s Disease Activity Index; quality of life; endoscopy; CRP; faecal calprotectin	Gastroparesis Cardinal Symptom Index; gastric emptying; nutrient tolerance
**Compound/Formulation** **Dose & Duration**	Oral CBD-rich cannabis oil (16% CBD, 4% THC) 8 weeks	Pharmaceutical-grade CBD (Epidiolex^®^) Up to 20 mg/kg/day for 4 weeks
**Population**	56 patients (34.5 ± 11 years), 30 men/26 women; 30 cases/26 placebo group	44 patients (32 idiopathic, 6 diabetes mellitus type 1, 6 diabetes mellitus type 2)
**Study Design**	Randomised, double-blind, placebo-controlled trial	Randomised, double-blind, placebo-controlled trial
**Disease**	Crohn’s disease	Idiopathic or diabetic gastroparesis

**Table 5:** Negative or Unsuccessful Clinical Outcomes with Cannabinoids.

Ref.	[127]	[128]	[129]	[130]	[131]
**Safety**	No major safety concerns highlighted	Acceptable tolerability (no major safety signals reported)	Well tolerated	No serious adverse events reported; no clinically meaningful next-day cognitive or driving impairment; mild increase in subjective sedation	No major safety issues reported
**Key Findings**	Modest, inconsistent behavioural improvements; no robust statistical significance; limited power	Failed to meet primary and secondary endpoints; no clinically meaningful benefit	No significant reduction in tremor or motor improvement vs. placebo	No significant differences in 27/28 cognitive and psychomotor tests vs. placebo; small reduction in Stroop–Colour accuracy (−1.4%, *p* = 0.016; likely not clinically meaningful); no impairment in simulated driving performance; small increase in subjective sedation at 10 h post-dose	No significant between-group differences in objective sleep outcomes; substantial placebo response
**Main Outcomes**	Behavioural scales (caregiver- and clinician-rated)	Behavioural functioning (primary endpoint)	Tremor amplitude (accelerometry); motor performance	Next-day cognitive performance; psychomotor function; simulated driving performance; subjective drug effects; mood	Actigraphy sleep parameters; subjective sleep quality
**Compound/Formulation** **Dose & Duration**	CBD 250 mg or 500 mg daily [weight-based] 12 weeks	CBD 250 mg or 500 mg daily [weight-based] 12 weeks	Single oral CBD 300 mg; Acute administration; two experimental sessions performed 2-weeks apart	Oral medicinal cannabis oil; 10 mg Δ9-THC + 200 mg CBD (1:20 THC:CBD ratio); suspended in medium-chain triglyceride (MCT); Single dose (10 mg THC + 200 mg CBD); administered 1 h before bedtime; outcomes assessed ≥9–10 h post-dose	Oral liquid dose of 30 mg CBN, 300 mg CBN
**Population**	212 patients, mean age 9.7 years, 75% males	240 patients; mean age 9.7 years (range 3–17 years); male (76.3%)	19 patients; 10 males/9 females; mean 63 years of age	20 adults; Mean age 46.1 ± 8.6 years; 16 females	20 adults
**Study Design**	Early-phase clinical study	Phase III randomised, double-blind, placebo-controlled trial	Randomised, double-blind, placebo-controlled crossover	Pilot randomized, double-blind, placebo-controlled, crossover trial; two 24-h in-laboratory visits; 1:1 randomization; ≥7-day washout	Placebo-controlled trial
**Disease**	Fragile X syndrome	Fragile X syndrome	Essential tremor	Insomnia disorder (DSM-5 criteria)	Insomnia
**Ref.**	**[132]**	**[133]**	**[134]**	**[135]**	**[136]**
**Safety**	No major safety signals described	Generally well tolerated; mostly mild adverse events; no signal of worsening suicidality	Acceptable tolerability; mostly mild adverse events; no serious adverse events attributed to CBD	No major safety concerns reported	Increased cognitive adverse events
**Key Findings**	Symptom reduction in both groups; no significant between-group differences; high placebo response	Reduction in depressive symptom severity compared to placebo; improvement observed in secondary anxiety measures; effect size in the small-to-moderate range	No significant additive benefit vs. placebo; no increased risk of manic switch observed; response/remission rates not significantly different between groups	No improvement vs. placebo across motor, cognitive, or inflammatory outcomes	Worsened semantic verbal fluency; higher subjective cognitive AEs
**Main Outcomes**	Anxiety and depression scales	Change in depressive symptoms, Anxiety symptoms, sleep measures, safety/tolerability assessments	Change in depressive symptoms (Montgomery–Åsberg Depression Rating Scale); Response and remission rates; anxiety symptoms; manic symptom monitoring; safety and tolerability	Cognition; MDS-UPDRS III; affective symptoms; inflammatory markers	Cognitive measures; verbal fluency
**Compound/Formulation** **Dose & Duration**	300 mg oral CBD; 3 and 6 months follow-up	Standard oral THC doses (5 mg); daily oral dose; 4–8 weeks	Highly purified pharmaceutical-grade CBD; daily oral solution as adjunctive therapy to ongoing mood stabilizers/antipsychotics; Initiated at 150 mg/day, titrated up to 300 mg/day based on tolerability/clinical response; 8 weeks	Sublingual CBD-enriched product (101.9 mg/mL CBD, 4.8 mg/mL THC); CBD 26 mg/day, THC 1.2 mg/day; 12 weeks	Oral CBD/THC (100 mg CBD/3.3 mg THC) 16.3 (SD: 4.2) days; dosage escalating to twice/day
**Population**	39 cases/41 placebos	*n* = 33; average age 40 years (range 20–66); 36% male/64% female	30 adults	51 participants (CBD: 27; placebo: 24)	58 patients
**Study Design**	Randomised controlled trial	Randomised controlled trial	Randomized, double-blind, placebo-controlled, parallel-group pilot trial; adjunctive design	Randomized, double-blind, placebo-controlled, parallel-group clinical trial	Randomized, double-blind, parallel-group, placebo-controlled study
**Disease**	Anxiety and depressive disorders	Anxiety and depressive disorders	Bipolar depression (adjunctive)	Parkinson’s disease	Parkinson’s disease
**Ref.**	**[137]**	**[110,138]**	**[110,138]**	**[139]**	**[140]**
**Safety**	No major safety signals reported	Well tolerated	Well tolerated	↑ systolic BP; transient delusions; hypertension; potential harm signal	Illustrates diagnostic risk rather than therapeutic effect
**Key Findings**	No superiority vs. placebo; some caregiver-reported domains favoured placebo	CBD did not reduce RBD manifestations in PD patients; No objective improvement on v-PSG; Temporary improvement in subjective sleep satisfaction	No improvement in sleep severity or objective parameters	CBD worsened delayed recall; greater increase in psychotic symptoms; 7 marked psychotic exacerbations	Cannabis use masked porphyria presentation, delaying diagnosis
**Main Outcomes**	Motor and non-motor outcomes	Transient improvement in sleep satisfaction at weeks 4 and 8 (CBD vs. placebo); No significant changes in motor, mood, anxiety, or polysomnography measures; No significant difference vs. placebo for RBD frequency; No significant difference in Clinical Global Impression—Severity and Improvement	Sleep scales; polysomnography	HVLT-R delayed recall; PANSS-P	Diagnostic course
**Compound/Formulation** **Dose & Duration**	Oral cannabis extract (up to 2.5 mg/kg/day) 2 weeks	CBD, 99.6% pure powder Oral capsules (corn oil) Dose escalation (week 1: 75 mg/day; week 2: 150 mg/day; weeks 3–12: 300 mg/day) Once daily after dinner	CBD, 75–300 mg 12 weeks	Single oral CBD 1000 mg prior to THC (20–60 mg inhaled)	Cannabis exposure (non-standardised)
**Population**	CBD/THC (*n* = 31)/placebo (*n* = 30)	33 Adults (mean age ~57 years)	18 adults (6 cases, 12 placebos)	30 patients (18–65 years)	Single patient
**Study Design**	Randomised trial	Phase II/III Randomized, double-blind, placebo-controlled, parallel-group trial	Phase II/III, parallel, double-blind, placebo-controlled clinical trial	Randomised, double-blind, placebo-controlled crossover	Case report
**Disease**	Parkinson’s disease	REM Sleep Behavior Disorder in Parkinson’s Disease	Restless Legs Syndrome/Willis–Ekbom Disease in patients with Parkinson’s disease and Rapid Eye Movement sleep behavior disorder	Schizophrenia with cannabis use disorder	Porphyria (diagnostic interference case)

Caption: ↑ increase.

## Data Availability

Not applicable.
